# Antioxidant and metabolic adjunctive treatment in late onset psychosis

**DOI:** 10.1192/j.eurpsy.2024.579

**Published:** 2024-08-27

**Authors:** T. Prokhorova, I. Boksha, O. Savushkina, E. Tereshkina, E. Vorobyeva, G. Burbaeva

**Affiliations:** FSBSI “MENTAL HEALTH RESEARCH CENTRE”, Moscow, Russian Federation

## Abstract

**Introduction:**

Basing on our previous findings of significant additional gain obtained from usage of adjunctive antioxidant medicine added to antipsychotic+antidepressant therapy in late-onset schizophrenia-like psychoses (LOP), the group often suffering of comorbid pathologies and experiencing substantial side-effects of drugs, we spred our approach to try “metabolic” medicines as adjunctives in LOP.

**Objectives:**

To reveal biochemical parameters of the blood cells which might be used for distinguishing subgroups of patients suffering with LOP for whom various adjunctive therapy (antioxidant, metabolic) would be advantageous.

**Methods:**

The study included 59 patients 50-89 years old, with LOP (onset after 40 years), and 38 healthy peoples 51 – 84 years old. The activities of glutamate dehydrogenase (GDH), glutathione reductase (GR), and glutathione S-transferase (GST) were determined spectrophotometrically in erythrocytes and platelets. Scores by PANSS were evaluated twice: before and on the 28-th day of antipsychotic treatment.

**Results:**

Samples from control group were used for determination of the control ranges for levels of studied enzymatic activities. Enzymatic activity levels were analyzed in three groups of patients: group Gr1 (n=16) treated without adjunctive therapy, and two other groups (Gr2 and Gr3) treated with adjunctive medicines: antioxidant 2-ethyl-6-methyl-3-hydroxypyridine succinate (Gr2, n=20), or “metabolic” medicines citicoline/cerebrolysin/cortexin/actovegin/gliatilin (Gr3, n=23).

As compared with controls, activity of erythrocyte GR was decreased at baseline and after the treatment course in all patients’ groups (p<0.01); in Gr2 significant decreases in baseline platelet GDH and GST activities were observed (p=0.005). Different significant links between biochemical parameters and scores by clinical scales before treatment were observed: in Gr1, erythrocyte GST activity positively correlated with scores by PANSS-Neg (R=0.61, p=0.012), by PANSS-Psy (R=0.54, p=0.032), and by PANSS (R=0.62, p=0.010), in Gr2, erythrocyte GST activity positively correlated with scores by PANSS-Pos (R=0.53, p=0.016), by PANSS-Psy (R=0.52, p=0.015), and by PANSS (R=0.60, p=0.005), in Gr3, platelet GR activity positively correlated with PANSS-Pos (R=0.50, p=0.014).

**Conclusions:**

We have confirmed the additional favor (decrease in side-effect severity) obtained by distinct patient groups when treated with adjunctive antioxidant or “metabolic” therapy. Moreover, correlations revealed in the patient subgroups between enzymatic activities and scores by psychometric scales enable revealing those biochemical markers measurement of which facilitate differentiating the patients for whom the adjunctive medicines to antipsychotic+antioxidant treatment can positively influence the treatment outcome.

**Disclosure of Interest:**

None Declared

